
JOURNAL INFORMATION
==============================
NLM Title Abbreviation: CPT Pharmacometrics Syst Pharmacol
Journal ID: CPT Pharmacometrics Syst Pharmacol
NLM Journal ID: 101580011
Journal ID: PSP4

CPT: Pharmacometrics & Systems Pharmacology

EISSN: 2163-8306
Publisher: Wiley

ARTICLE INFORMATION
==============================
PMCID: PMC11474496
DOI: 10.1002/psp4.12988
Article ID: PSP412988
Article version: 1
Subjects: Issue Information, Issue Information

Issue Information

Electronic publication date: 2024 May 16
Publication date: 2024 May
Volume: 13
Issue: 5
First page: 687
Last page: 690
Copyright: © 2024 ASCPT
License: This is an open access article under the terms of the http://creativecommons.org/licenses/by/4.0/ License, which permits use, distribution and reproduction in any medium, provided the original work is properly cited.
License URL: https://creativecommons.org/licenses/by/4.0/

Figure count: 0
Table count: 0
Page count: 3

source-schema-version-number: 2.0
cover-date: May 2024
details-of-publishers-convertor: Converter:WILEY_ML3GV2_TO_JATSPMC version:6.4.9 mode:remove_FC converted:15.10.2024

==============================

An Official Journal of ASCPT and ISoP

### CPT: Pharmacometrics & Systems Pharmacology

Open Access

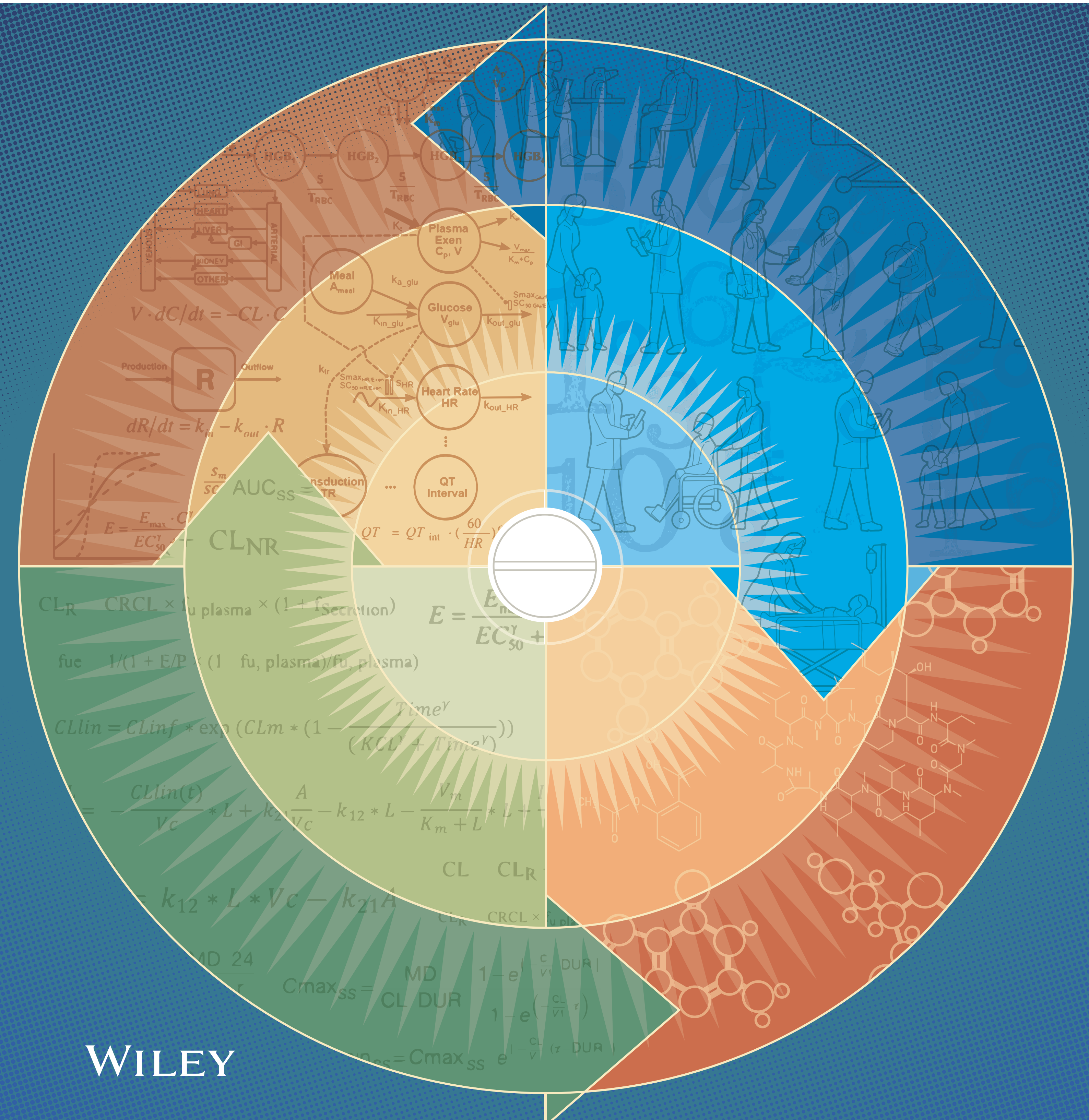

WILEY

CPT: Pharmacometrics & Systems Pharmacology

WILEY

**Editor-in-Chief**

France Mentré, MD, PhD, *School of Medicine of University Paris Diderot, Paris, France*

**Deputy Editor-in-Chief**

Karen Rowland Yeo, PhD, *Certara UK Ltd., Sheffield, UK*

**Associate Editors**

Paolo Denti, PhD, *University of Cape Town, Cape Town, South Africa*
Rajat Desikan, PhD, *GSK, Stevenage, UK*
Jonathan French, ScD, *Johnson & Johnson Innovative Medicine, Spring House, PA, USA*
Stefanie Hennig, MSc, PhD, *Certara Strategic Consulting, Brisbane, Australia*
Priya Jayachandran, PharmD, *Regeneron Pharmaceuticals, Inc., San Francisco, CA, USA*
Andrew Hooker, PhD, *Uppsala University, Uppsala, Sweden*
Sriram Krishnaswami, PhD, *Pfizer, Groton, CT, USA*
Ping Zhao, PhD, *Bill & Melinda Gates Foundation, Seattle, WA, USA*

**Editors-in-Training**

Brian Cicali, PhD, *University of Florida, College of Pharmacy, Orlando, FL, USA*
Jane Knöchel, PhD, *AstraZeneca , Mölndal, Sweden*

**American Society for Clinical Pharmacology and Therapeutics**

***Chief Executive Officer***
Abigail W. Gorman, *Alexandria, VA*
***Chief Operating Officer***
Elise S. Laffman-Johnson, *Alexandria, VA*
***Managing Editor***
Alethea B. Gerding, *Alexandria, VA*
***Deputy Managing Editor***
Alaina Webster, *Alexandria, VA*
***Senior Editorial Coordinator***
Aimee Greene, *Alexandria, VA*

**Editorial Board**

Vivaswath S. Ayyar, PhD, *Janssen Research & Development, Spring House, PA*
Robert Bies, PharmD, PhD, *State University of New York at Buffalo, Buffalo, NY, USA*
Marc Birtwistle, PhD, *Clemson University Clemson, SC, USA*
Yuan Chen, PhD, *Genentech South San Francisco, CA, USA*
Marylore Chenel, PharmD, PhD, *Pharmetheus Uppsala, Sweden*
Emmanuel Chigutsa, PhD, *Eli Lilly and Company, Indianapolis, IN, USA*
Teresa Dalla Costa, PhD, *Federal University of Rio Grande do Sul - UFRGS Porto Alegre, Brazil*
Narendra M Dixit, PhD, *Indian Institute of Science, Bengaluru, India*
Anne-Gaelle Dosne, PharmD, PhD, *Janssen Research & Development, Beerse, Belgium*
Thomas Dumortier, Eng, MSc, *Novartis Pharma AG, Basel, Switzerland*
James M. Gallo, PharmD, PhD, *University at Buffalo, Buffalo, NY, USA*
Wei Gao, PhD, *EMD Serono, Billerica, MA, USA*
Jérémie Guedj, PhD, *INSERM, Paris, France*
Manish Gupta, PhD, FCP, *GenMab; Princeton, NJ, USA*
Bart Hendriks, PhD, *Novartis Institutes for BioMedical Research Cambridge, MA, USA*
Nick Holford, MBChB, *The University of Auckland Auckland, New Zealand*
Brett A. Howell, PhD, *DILIsym Services Division, Simulations Plus, Inc., Research Triangle Park, NC, USA*
Manuel Ibarra, PhD, *Universidad de la República, Montevideo, Uruguay*
Siv Jönsson, PhD, *Uppsala University Sweden, Uppsala, Sweden*
Kristin Karlsson, PhD, *Uppsala University Uppsala, Sweden*
Helen Kastrissios, PhD, *QuanTx Consulting, Brisbane, Australia*
Ron Keizer, PhD, *InsightRX San Francisco, CA, USA*
Steven E. Kern, PhD, *Global Health Labs, Bellevue, WA, USA*
Akash Khandelwal PhD, *UCB Biosciences GmbH, Monheim am Rhein, Germany*
Andrzej Kierzek, PhD, *Certara QSP; Sheffield, United Kingdom*
Qi Liu, PhD, *US Food and Drug Administration, Silver Spring, MD*
James Lu, PhD, *Genentech, Inc., South San Francisco, CA, USA*
Rajanikanth (Raj) Madabushi, PhD, *US Food and Drug Administration, Silver Spring, MD*
Efthymios Manolis, MSc, *European Medicines Agency London, United Kingdom*
Samer Mouksassi, PharmD, PhD, *Certara, Cairo, Egypt*
Jackson K. Mukonzo, PhD, *Makerere University, Kampala Uganda*
Joakim Nyberg, PhD, *Pharmetheus, Uppsala, Sweden*
Elsje Pienaar, PhD, *Weldon School of Biomedical Engineering, Purdue University, West Lafayette, IN, USA*
Daniel Scholes Rosenbloom, PhD, *Merck & Co., Rahway, NJ, USA*
Joe Standing, PhD, *University College London London, United Kingdom*
Natasha Strydom, PhD, *Amgen, Inc., San Francisco, CA, USA*
Yuichi Sugiyama, PhD, *Riken Innovation Center Sugiyama Laboratory Yokohama, Japan*
Elin Svensson, PhD, *Uppsala University, Uppsala, Sweden*
Joel Tarning, PhD, *Mahidol-Oxford Tropical Medicine Research Unit Bangkok, Thailand*
Nadia Terranova, PhD, *Merck KGaA, Lausanne, Switzerland*
Michael Tortorici, PharmD, PhD, *Aro Biotherapeutics, Philadelphia, PA, USA*
Coen van Hasselt, PhD, *Leiden University, Leiden, the Netherlands*
Paolo Vicini, PhD, MBA, *Confo Therapeutics, Ghent, Belgium*
Camille Vong, PhD, *Novartis AG Pharma, Basel, Switzerland*
Xinyuan (Susie) Zhang, PhD, *Daiichi Sankyo, Inc. Basking Ridge, NJ, USA*

### Overview

*CPT: Pharmacometrics & Systems Pharmacology* (PSP) is an official open access journal of the American Society for Clinical Pharmacology and Therapeutics (ASCPT) and International Society of Pharmacometrics (ISoP).

*CPT: Pharmacometrics & Systems Pharmacology* is a cross-disciplinary journal devoted to publishing advances in quantitative (e.g., modeling and simulation) methods as applied in pharmacology, physiology and therapeutics in humans. The journal welcomes original research articles, reviews and tutorials that bridge the following areas: pharmacometrics, modeling and simulation as applied to the design and evaluation of clinical trials, systems pharmacology modeling, particularly with a mechanistic link to human (patho)physiology, disease modeling, "population" or mixed-effects pharmacokinetics and pharmacodynamics (PKPD) modeling, modeling and simulation to support translational research, physiologically-based pharmacokinetics (PBPK), model-based meta-analyses of clinical trials, mechanism-based pharmacokinetic-pharmacodynamic modeling, computational pharmacology, bioinformatics, comparative efficacy, effectiveness and cost-effectiveness. Systems pharmacology may involve the application of systems biology approaches to study drug activities, targets and effects. The discipline is often defined with reference to engineering and pharmacological principles as the quantitative analysis of the dynamic interactions between drugs and a biologic system that aims to understand the behavior of the system as a whole. The common focus will be on quantitative methods that improve our understanding of pharmacology and therapeutics in humans.

**Abstracting and Indexing**
- Embase (Elsevier)
- SCOPUS (Elsevier)
- MEDLINE/PubMed indexing

**Society Information**
The American Society for Clinical Pharmacology and Therapeutics (ASCPT) was founded in 1900, and consists of over 2,200 professionals whose primary interest is to advance the science and practice of clinical pharmacology and translational medicine for the therapeutic benefit of patients and society. ASCPT is the largest scientific and professional organization serving the disciplines of Clinical Pharmacology and Translational Medicine.

**All articles published in this journal are freely available to read, download, and share. Articles are published under the terms of the Creative Commons Attribution Non-Commercial License which permits use, distribution, and reproduction in any medium, provided the original work is properly cited and is not used for commercial purposes.**

**Disclaimer**
The Publisher, the American Society for Clinical Pharmacology and Therapeutics, the International Society of Pharmacometrics, and Editors cannot be held responsible for any errors in or any consequences arising from the use of information contained in this journal. The views and opinions expressed do not necessarily reflect those of the Publisher, the American Society for Clinical Pharmacology and Therapeutics, the International Society of Pharmacometrics or Editors, neither does the publication of advertisements constitute any endorsement by the Publisher, the American Society for Clinical Pharmacology and Therapeutics, the International Society of Pharmacometrics, Editors, or Authors of the products advertised.

**Publisher**
*CPT: Pharmacometrics & Systems Pharmacology* is published by Wiley Periodicals, LLC, 101 Station Landing, Suite 300, Medford, MA 02155; Telephone: 781-388-8200; Fax: 781-388-8210.

### CONTENTS

CPT: Pharmacometrics & Systems Pharmacology

### REVIEWS

REVIEWS

691 Realizing the promise of Project Optimus: Challenges and emerging opportunities for dose optimization in oncology drug development
**Wei Gao, Jiang Liu, Blerta Shtylla, Karthik Venkatakrishnan, Donghua Yin, Mirat Shah, Timothy Nicholas and Yanguang Cao**

710 Covariate modeling in pharmacometrics: General points for consideration
**Kinjal Sanghavi, Jakob Ribbing, James A. Rogers, Mariam A. Ahmed, Mats O. Karlsson, Nick Holford, Estelle Chasseloup, Malidi Ahamadi, Kenneth G. Kowalski, Susan Cole, Essam Kerwash, Janet R. Wade, Chao Liu, Yaning Wang, Mirjam N. Trame, Hao Zhu, Justin J. Wilkins and for the ISoP Standards & Best Practices Committee**

SYSTEMATIC REVIEW

729 Model-based meta-analysis to quantify the effects of short interfering RNA therapeutics on hepatitis B surface antigen turnover in hepatitis B-infected mice
**Louis Sandra, Huybrecht T'jollyn, An Vermeulen, Oliver Ackaert and Juan-Jose Perez-Ruixo**

### TUTORIAL

TUTORIAL

743 Using forest plots to interpret covariate effects in pharmacometric models
**E. Niclas Jonsson and Joakim Nyberg**

### RESEARCH

ARTICLES

759 RPEM: Randomized Monte Carlo parametric expectation maximization algorithm
**Rong Chen, Alan Schumitzky, Alona Kryshchenko, Keith Nieforth, Michael Tomashevskiy, Shuhua Hu, Romain Garreau, Julian Otalvaro, Walter Yamada and Michael N. Neely**

781 Development of a physiologically-based pharmacokinetic model to simulate the pharmacokinetics of intramuscular antiretroviral drugs
**Sara Bettonte, Mattia Berton, Manuel Battegay, Felix Stader and Catia Marzolini**

795 Medication adherence framework: A population-based pharmacokinetic approach and its application in antimalarial treatment assessments
**Junjie Ding, Richard M. Hoglund and Joel Tarning**

812 Evaluation of covariate effects in item response theory models
**Gustaf J. Wellhagen, Ashraf Yassen, Dirk Garmann, Astrid Bröker, Alexander Solms, Yang Zhang, Maria C. Kjellsson and Mats O. Karlsson**

823 Pharmacokinetics and exposure–safety relationship of ciprofol for sedation in mechanically ventilated patients in the intensive care unit
**Lu Liu, Kun Wang, Zhongyi Sun, Pangke Yan, Mengyue Hu, Xiao Liu, Meixia Chen, Nan Wu and Xiaoqiang Xiang**

837 Construction of a prognostic model based on memory CD4+ T cell–associated genes for lung adenocarcinoma and its applications in immunotherapy
**Yong Li, Xiangli Ye, Huiqin Huang, Rongxiang Cao, Feijian Huang and Limin Chen**

853 A physiologically-based pharmacokinetic/pharmacodynamic modeling approach for drug–drug-gene interaction evaluation of S-warfarin with fluconazole
**Kuo Geng, Chaozhuang Shen, Xiaohu Wang, Xingwen Wang, Wenxin Shao, Wenhui Wang, Tao Chen, Hua Sun and Haitang Xie**

870 Deep-NCA: A deep learning methodology for performing noncompartmental analysis of pharmacokinetic data
**Gengbo Liu, Logan Brooks, John Canty, Dan Lu, Jin Y. Jin and James Lu**

880 Item response theory analysis of daytime sleepiness as a symptom of obstructive sleep apnea
**Gustaf J. Wellhagen, Mats O. Karlsson, Maria C. Kjellsson, Dirk Garmann, Astrid Bröker, Yang Zhang, Mika Nokela, Gerrit Weimann and Ashraf Yassen**

891 Population longitudinal analysis of Gait Profile Score and North Star Ambulatory Assessment in children with Duchenne muscular dystrophy
**Jiexin Deng, Fangli Liu, Zhifen Feng and Zhigang Liu**
